# High IgG4 serum concentration is associated with active Graves orbitopathy

**DOI:** 10.3389/fendo.2023.1083321

**Published:** 2023-03-01

**Authors:** Michał Olejarz, Ewelina Szczepanek-Parulska, Anna Ostałowska-Klockiewicz, Patrycja Antosik, Nadia Sawicka-Gutaj, Celina Helak-Łapaj, Marcin Stopa, Marek Ruchala

**Affiliations:** ^1^ Department of Endocrinology, Metabolism and Internal Medicine, Poznan University of Medical Sciences, Poznań, Poland; ^2^ Department of Ophthalmology, Chair of Ophthalmology and Optometry, Poznan University of Medical Sciences, Poznan, Poland

**Keywords:** IgG4, Graves disease, Graves orbitopathy, ophtalmopathy, IgG4-related disease, thyroid eye disease (TED)

## Abstract

**Background:**

The aim of the study was to evaluate the differences in clinical profile, laboratory parameters, and ophthalmological signs, and symptoms between patients with high IgG4 Graves orbitopathy and patients with normal IgG4 Graves orbitopathy.

**Methods:**

This was a prospective observational study. We recruited adult patients with Graves Orbitopathy(GO) referred to our clinic for further diagnostics and treatment. Eventually, 60 patients with GO were enrolled in the study. All patients underwent ophthalmological assessment, magnetic resonance imaging (MRI) of the orbits, and laboratory tests, including IgG4 serum concentration measurement. High IgG4 GO was diagnosed if the IgG4 concentration exceeded 135 mg/dl. We used both the clinical activity score (CAS) and magnetic resonance imaging (MRI) to assess the activity of GO. Eventually, active GO was defined according to MRI results.

**Results:**

Among 60 GO patients, 15 (25%) patients had elevated IgG4 levels. Patients in the high IgG4 group had a higher prevalence of active GO by MRI than patients with normal IgG4 (100% vs. 64.44%, P=0.006). They also had a higher eosinophile count in peripheral blood, a lower bilirubin level, a more frequent lower eyelid retraction, and a lower prevalence of glaucoma. There were no statistically significant differences between the groups in CAS. Patients with active GO, had higher median IgG4 level [89.95 (55.48; 171.1) vs 43.45 (32.48; 49.68) mg/dl, P<0.001]. The receiver operating characteristic (ROC) analysis for IgG4 as a marker of active GO revealed the following results: AUC 0.848 for the cut-off value of 54.2 mg/dl, sensitivity 79.5%, specificity 87.5%, positive predictive value 94.6%, negative predictive value 59.1%.

**Conclusions:**

We demonstrated that IgG4 is a marker of GO activity. Certain differences in the clinical profile of patients with high IgG4 GO, and normal IgG4 GO were observed. More data is needed to establish whether patients with high IgG4 GO are GO patients with particularly active disease or actually represent a distinct clinical entity related to IgG4-Related Disease.

## Introduction

Human immunoglobulin G4 (IgG4) usually constitutes less than five percent of the total amount of IgG in the serum. This makes it the least abundant IgG subclass but certainly not the least important (1). It has unusual biological features that distinguish it from other antibodies, such as inhibition of the formation of immune complexes, lack of antibody-dependent cell-mediated cytotoxicity, and the capability of undergoing Fab-arm exchange (2). Its role in the inflammatory process can be protective (e.g., immune tolerance-inducing), directly pathogenic, or it could be a marker of an atypical inflammatory response (3). IgG4 has been the focal point of many research projects in recent years due to its unique characteristics. High serum concentrations of IgG4 were firstly described in a group of patients with sclerosing autoimmune pancreatitis by Hamano et al. (4). This further led to the discovery of IgG4-related disease (IgG4-RD), which is a chronic disorder characterized by fibrosis and inflammation. Typical features of IgG4-RD are increased plasma level of IgG4, tissue infiltration with IgG4-positive plasma cells, obliterative phlebitis, storiform fibrosis, and a very good response to glucocorticoid treatment (5–7). The disease affects predominately white males in their middle age or older. However, interestingly, patients with the disease limited to the head and neck region were more commonly females, especially of Asian origin (7–9).

The course of IgG4-RD can be heterogeneous and depends on the affected area. It usually affects the pancreas, salivary and lacrimal glands, lungs, biliary tract, retroperitoneum, kidneys, liver, or even aorta^3,6^. Typical manifestations are pancreatitis, chronic sclerosing sialadenitis, dacryoadenitis, thyroiditis, mediastinal or retroperitoneal fibrosis, periaortitis, cholangitis, inflammatory pseudotumor, and Mikulicz disease (7, 10).

The role of IgG4 has also been evaluated in the thyroid disease. Based on current research, the fibrosing variant of Hashimoto’s thyroiditis and Riedel’s thyroiditis show the biggest histological and clinical resemblance to IgG4-RD. They are acknowledged by many as disorders of the IgG4-RD spectrum. However, high levels of IgG4 have also been noticed in some patients with Graves disease (GD), especially in a subgroup of patients with Graves orbitopathy (GO) (11). Graves orbitopathy is the most common extrathyroidal manifestation of Graves disease (GD). Despite ongoing research and development of new treatment methods, managing GO still poses several diagnostic and therapeutic challenges (12, 13). Thus, the development of new diagnostic tools is crucial.

So far, the role of IgG4 in GD and orbitopathy has been assessed to a limited extent. According to a recent review, the average prevalence of elevated IgG4 levels in GD patients is around 10% (5.4% in patients without GO and 17.6% in patients with orbitopathy). While some studies linked elevated IgG4 levels with the occurrence of GO and its activity, better response to thiamazole, or increased thyroid antibody levels, other studies failed to find such associations (14, 15). The vast majority of studies were carried out on the Asian population, while data from other regions are scarce. Moreover, several study groups included only a limited number of patients with GO in their investigation.

In our study, we aimed to evaluate whether IgG4 can be used as a marker of GO activity in clinical practice and assess the differences in clinical profile, laboratory parameters, and ophthalmological signs and symptoms between patients with GO and high IgG4 serum concentration and patients with GO and normal IgG4 serum concentration.

## Materials and methods

### Study design and patients’ enrollment

This was a prospective observational study. It was conducted at the Department of Endocrinology, Metabolism, and Internal Medicine of the Poznan University of Medical Sciences (tertiary referral hospital). We recruited all adult patients (>= 18 years old) diagnosed with Graves orbitopathy, who were referred to our department for diagnostics and treatment of the disease. Exclusion criteria were: 1) systemic immunosuppressive or immunomodulatory treatment at the time of admission or in the previous 6 months; 2) Chronic kidney disease stages 4 and 5; 3) Liver failure; 4) Active neoplastic disease or suspicion of malignant disease; 5) Active acute or chronic infections; 6) Any immunodeficiency disorder 7) pregnancy.

Eventually, 60 patients with GO were enrolled in the study, of whom 25 underwent previous thyroidectomy, 28 were treated with radioiodine in the past (5 underwent both thyroidectomy and radioiodine therapy), and 6 were still on antithyroid medication. All patients were screened for human immunodeficiency virus, hepatitis B, and hepatitis C. Patients who were carriers of those viruses were excluded from the study.

A detailed history of treatment and comorbidities, including smoking status, was obtained from each patient.

### Ophthalmologic examination

An extensive ophthalmologic examination was performed in each patient by an experienced ophthalmologist who was blinded to IgG4 results throughout the study. Hertl’s exophthalmometer was used for precise exophthalmos measurements. Patients were evaluated according to the Clinical Activity Score (CAS), based on the following symptoms 1) Periorbital or retroorbital pain or pressure; 2) Pain with lateral, downward, or upward movement, and following signs 1) Swelling of the eyelids; 2) Redness of the eyelids; 3) Conjunctival injection; 4) Chemosis; 5) Inflammation of the caruncle or plica. Intraocular pressure (IOP) values were obtained in each patient with Goldmann applanation tonometry. IOP values over 21 mm Hg were considered diagnostic for glaucoma. However, it is worth noting that some patients have been diagnosed with glaucoma before entering the study and at the time of admission had IOP <21 on IOP lowering medication. Patient’s best corrected visual acuity was measured and defined according to the WHO definitions of vision impairment: mild – visual acuity worse than 6/12 to 6/18; moderate – visual acuity between 6/18 and 6/60; severe – visual acuity between 6/60 and 3/60, blindness – visual acuity worse than 3/60. Extraocular muscle involvement was defined as restriction in eyeball motility resulting in double vision in either primary or extreme gaze positions. Optic nerve involvement was defined as pallor or choking of the optic disc with preserved or diminished visual acuity; Corneal involvement was defined as the presence of stippling, ulceration, clouding, or perforation of the cornea.

### Laboratory assessment

Fasting (overnight) venous blood samples were taken from each patient. Detailed laboratory analysis was conducted, including thyroid-related hormones [thyroid-stimulating hormone (TSH), free triiodothyronine (fT3), free thyroxine (fT4)), thyroid autoantibody profile (anti-TSH receptor antibodies(TRAb), anti-thyroid peroxidase antibodies (TPOAb), anti-thyroglobulin antibodies (TgAb)], renal function (creatinine), liver function (ALT, AST, bilirubin), electrolytes (total calcium, sodium, potassium), metabolic profile (total cholesterol, HDL cholesterol, LDL cholesterol, triglycerides) and inflammatory process markers (C-reactive protein) and complete blood count with differential (CBC-D).

The CBC-D was measured by the flow cytometry based hematology analyzer Sysmex-XN 1000 (Sysmex Europe GmbH) TRAb levels were evaluated by the radioimmunological method with a commercially available radioimmunoassay kit (Brahms GmbH). The rest of the parameters mentioned above were assessed with the use of the COBAS 8000 analyzer (Roche).

Finally, IgG4 serum concentration was measured by an immune–enzymatic assay using commercially available ELISA kits (Sunredbio, Shanghai). Based on IgG4 levels, we divided patients into two groups – GO with high IgG4 and GO with normal IgG4 serum concentration. We applied the widely recognized cut-off value diagnostic for IgG4-RD (135 mg/dl), which was also used by the majority of previous studies on the role of IgG4 in GD.

### Imaging tests

Each patient underwent thyroid ultrasound. The volume of each thyroid lobe was calculated with the use of the following formula: 0.52 x length x width x depth. The cumulative volume of the thyroid gland was obtained after adding together volumes of the right and left lobe. For differential diagnosis and to assess the activity of GO, we performed a magnetic resonance imaging (MRI) of the orbits in each patient. MRI was performed by Magnetom Skyra 3T scanner(Siemens Healthcare Gmbh). Patients with signs of active inflammatory process in dedicated sequence of the MRI of the orbits were placed in the active GO group, while patients without signs of active inflammation were placed in the inactive GO group. Active disease was diagnosed if the MRI revealed swollen extraocular muscles with increased signal intensity in the STIR(short tau inversion recovery) or T2-weighted sequences. If the imaging test did not show any abnormalities or just swelling of the extraocular muscles without increased signal intensity then inactive GO was diagnosed.

### Ethics

Every patient signed a written informed consent to participate in the study. The project was conducted in accordance with the declaration of Helsinki (16). The study was reviewed and approved by the Bioethics Committee at the Poznan University of Medical Sciences (Decision number: 774/20)

### Statistical analysis

The statistical analyses were performed using PQ Stat v.1.8.4. The Shapiro-Wilk test was used to check for normality. We assessed the equality of variances with Levene’s test. We used the unpaired student’s T-test to compare continuous, normally distributed data with homogenous variances. The Mann-Whitney U test was used to compare nonparametric data. The comparison of qualitative variables was carried out with the use of Fisher’s exact test. Pearson’s test was used for the correlation analysis of parametric data, while the Spearman test was used for the correlation analysis of nonparametric data. Data are presented as mean ± Standard Error (SE) or median (first Quartile; third Quartile). The P level <0.05 was considered significant. All tests were two-tailed. The ROC (receiver operating characteristic) curves were built. AUC was calculated with the DeLong method. The P level <0.05 was considered significant. All tests were two-tailed.

## Results

A total number of 60 patients were enrolled in the study. We divided patients into the high IgG4 group (IgG4 serum concentration >135 mg/dl) and normal IgG4 group (IgG4 serum concentration <135 mg/dl). 15 (25%) patients were placed in the high IgG4 group and 45 (75%) in the normal IgG4 group. Patients did not differ significantly in age, gender, or smoking status. There were also no statistically significant differences between those two groups in the number of patients treated with levothyroxine or antithyroid medications. The groups also did not differ in the number of patients who previously were treated with thyroidectomy radioiodine or received intravenous glucocorticoid pulse therapy in the past (>6 months ago). The thyroid volume was comparable among those two groups (a separate analysis was done for patients who received radioiodine in the past). There was no statistically significant difference in IgG4 serum concentrations between patients previously treated with methylprednisolone pulse therapy compared to patients who have never been treated (98.65 (48.53; 156.4) vs. 60.3 (47.43; 119.83) mg/dl, P=0.275). A detailed summary of patient’s ultrasound results, history and demographic data are presented in **Table 1**.

**Table 1:** Summary of patient history and demographic data.

	Normal IgG4 group (n=45)	High IgG4 group (n=15)	P value
Age (years)	53.93 ± 16.71	54.27 ± 14.29	0.946
Time since Graves orbitopathy onset (months)	18 [11; 36]	12 [5; 36]	0.328
Gender [n of males(%)]	10 (22.22%)	4 (26.67%)	0.734
Prior thyroidectomy	21 (46.67%)	4 (26.67%)	0.232
Prior radioiodine therapy	20 (44.44%)	8 (53.33%)	0.567
Prior intravenous glucocorticoid pulse therapy >6 months ago	10 (23.91%)	4 (28.57%)	0.733
No thyroidectomy or radioiodine	8 (17.78%)	4 (26.67%)	0.472
Levothyroxine therapy	39 (86.67%)	11 (73.33%)	0.250
Antithyroid treatment	3 (6.67%)	3 (20%)	0.159
Active smoker	22 (48.89%)	5 (33.33%)	0.375
Past smoker	4 (8.89%)	0 (0%)	0.564
Thyroid volume in patients after radioiodine treatment (cm^3^)	3.38 ± 0.77	2.10 ± 0.72	0.325
Thyroid volume in patients with an intact thyroid gland (cm^3^)	27.20 ± 7.33	21.69 ± 9.25	0.656

Continuous variables with a normal distribution are shown as mean ± SE, nonparametric variables are shown as median [lower quartile, upper quartile], nominal variables are snown as N (%).

Patients in the high IgG4 group had a higher prevalence of active GO than patients in the normal IgG4 group (100% vs. 64.44%, P=0.006). Patients in the elevated IgG4 group had also a higher eosinophile count (240 (190–320) vs 130 (70–250) cells/μl, P=0.041) and a lower total bilirubin level [0.49 (0.4; 0.59) vs 0,67 (0.51; 0.59) mg/dl, P=0.019]. Other biochemical parameters, including complete blood count, TRAb, TPOAb, TgAb, TSH, fT3, fT4, ALT, AST, creatinine, electrolytes, and lipid profile parameters, did not differ significantly. A full comparison of biochemical parameters is presented in **Table 2**.

**Table 2:** Comparison of laboratory parameters between the high IgG4 group and normal IgG4 group.

Parameter	Normal IgG4 group (n=45)	High IgG4 group (n=15)	P value
TSH (μU/ml)	1.4 [0.58; 3.75]	0.97 [0.35; 1.89]	0.338
fT3 (pmol/l)	4.19 [3.57; 4.67]	4.61 [3.85; 5.38]	0.156
fT4 (pmol/l)	18.6 [16; 20.4]	19.1 [13.85; 21]	0.880
TRAb (IU/I)	3.81 [1.73; 19.11]	9.66 [3.76; 12.49]	0.920
TPOAb (IU/ml)	82 [12.5; 205]	34 [15.75; 104]	0.363
TgAb (IU/ml)	15.5 [13; 147]	17 [13.5; 849]	0.600
Creatinine (mg/dl)	0.77 [0.65; 0.88]	0.72 [0.69; 0.81]	0.814
Total calcium (mg/dl)	9.71 [9.39; 9.94]	9.74 [9.43; 10.11]	0.592
Sodium (mmol/l)	141 [139; 142]	141 [138.5; 142.75]	0.739
Potassium (mmol/l)	4.36 [4.24; 4.71]	4.44 [4.3; 4.75]	0.563
Bilirubin (mg/dl)	0.67 [0.51; 0.79]	0.49 [0.4; 0.59]	**0.019**
AST (U/I)	19 [16; 24]	21 [17; 26.5]	0.316
ALT (U/I)	16 [13; 24]	18 [15.5; 25]	0.392
Glucose (mg/dl)	95 [90.75; 109.5]	98 [93; 104.5]	0.864
Total Cholesterol (mg/dl)	204.27 ± 66.14	205.93 ± 44.12	0.952
HDL (mg/dl)	61 [52; 69]	58 [52; 66.5]	0.632
LDL (mg/dl)	131.65 ± 53.5	134.31 ± 37.92	0.869
Triglycerides (mg/dl)	123,5 [93.25; 152.5]	113 [93; 130]	0.660
WBC (x10^3/μl)	6.37 [5.19; 7.41]	5.87 [5.13; 7.71]	0.951
Neutrophiles (x10^3/μl)	3.13 [2.54; 3.92]	3.32 [2.86; 3.85]	0.814
Lymphocytes (x10^3/μl)	2.11 ± 0.72	2.28 ± 0.69	0.568
Monocythes (x10^3/μl)	0.53 [0.4; 0.58]	0.42 [0.33; 0.51]	0.236
Eosinophiles (x10^3/μl)	0.13 [0.07; 0.25]	0.24 [0.19; 0.32]	**0.041**
RBC (x10^6/μl)	4.53 ± 0.4	4.68 ± 0.41	0.210
Hemoglobin (g/dl)	13.89 ± 1.48	14.19 ± 1.24	0.487
Platelets (x10^3/μl)	251.95 ± 66.42	248.53 ± 61.75	0.862
CRP (mg/l)	1.5 [0.7; 2.6]	1.8 [0.65; 3.75]	0.691
IgG4 (mg/dl)	51.9 [39.5; 82.9]	236.3 [172.3; 308.75]	**<0.001**

Statistically significant P values (<0.05) were displayed in bold.

We performed a detailed ophthalmologic examination to check for differences in ocular signs and symptoms between the high IgG4 and normal IgG4 groups. Patients in the high IgG4 group had more frequently lower eyelid retraction (93.33% vs. 62.22%, P=0.025). They suffered less often from glaucoma (51.11% vs. 20%, P=0.041). We also observed that patients with elevated IgG4 levels tended to manifest conjunctival injection more frequently (60% vs. 28.89%), but the result failed to achieve statistical significance (P=0.060). There were no statistically significant differences between the groups in CAS (and its components), intraocular pressure (IOP), visual acuity, proptosis, soft tissue involvement, extraocular muscle involvement, optic nerve involvement, GO signs (Dalrymple’s, Kocher’s, Rosenbach’s, Von Graefe’s, Möbius). Findings from the extensive ophthalmological examination are summarized in **Table 3**.

**Table 3:** Comparison of findings from ophthalmological examination in the normal and high IgG4 groups.

	Normal IgG4 group (n=45)	High IgG4 group (n=15)	P value
Active phase of orbitopathy	29 (64.44%)	15 (100%)	**0.006**
IOP in the right eye (mm Hg)	18.91 ± 3.32	18.2 ± 2.65	0.460
IOP in left eye (mm Hg)	18.47 ± 2.83	18.07 ± 4.16	0.690
Protrusion of the right eye (mm)	19.86 ± 4.23	20.43 ± 4.47	0.668
Protrusion of the left eye (mm)	19.57 ± 4.03	21.71 ± 3.81	0.087
Protrusion of the eye with greater proptosis (mm)	20.58 ± 3.98	21.93 ± 3.73	0.269
Lower eyelid retraction	28 (62.22%)	14 (93.33%)	**0.025**
Upper eyelid retraction	27 (60%)	9 (60%)	1
Soft tissue involvement	33 (73.33%)	14 (93.33%)	0.153
Proptosis	29 (64.44%)	9 (60%)	0.766
Extraocular muscle involvement	21 (46.67%)	7 (46.67%)	1
Corneal involvement	10 (22.22%)	2 (13.33%)	0.712
Optic nerve involvement	9 (20%)	1 (6.67%)	0.426
Glaucoma	23 (51.11%)	3 (20%)	**0.041**
Dalrymple’s sign	28 (62.22%)	12 (80%)	0.343
Kocher’s sign	12 (26.67%)	4 (26.67%)	1
Rosenbach’s sign	24 (53.33%)	7 (46.67%)	0.769
Von Graefe’s sign	17 (37.78%)	8 (53.33%)	0.369
Möbius sign	26 (57.78%)	5 (33.33%)	0.139
Pain or pressure in a periorbital or retroorbital distribution	13 (28.89%)	3 (20%)	0.738
Pain with upward, downward, or lateral eye movement	17 (37.78%)	3 (20%)	0.343
Redness of the eyelids	10 (22.22%)	3 (20%)	1
Conjunctival injection	13 (28.89%)	9 (60%)	0.06
Chemosis	10 (22.22%)	4 (26.67%)	0.734
Inflammation of the caruncle or plica	8 (17.78%)	5 (33.33%)	0.279
Swelling of the eyelids	25 (55.56%)	10 (66.67%)	0.552
CAS >=3	15 (33.33%)	7 (46.67%)	0.372
Mild or no vision impairment	41 (91.11%)	14 (93.33%)	1
Moderate to severe vision impairment or blindness	4 (8.89%)	1 (6.67%)	1

IOP, Intraocular pressure; CAS, Clinical Activity Score. Continuous variables with a normal distribution are shown as mean ± SE, nonparametric variables are shown as median [lower quartile, upper quartile], nominal variables are snown as N(%). Best corrected visual acuity was defined according to WHO definitions of vision impairment(blindness, moderate, severe, mild or no vision impairment). Statistically significant P values (<0.05) were displayed in bold.

**Figure 1:**
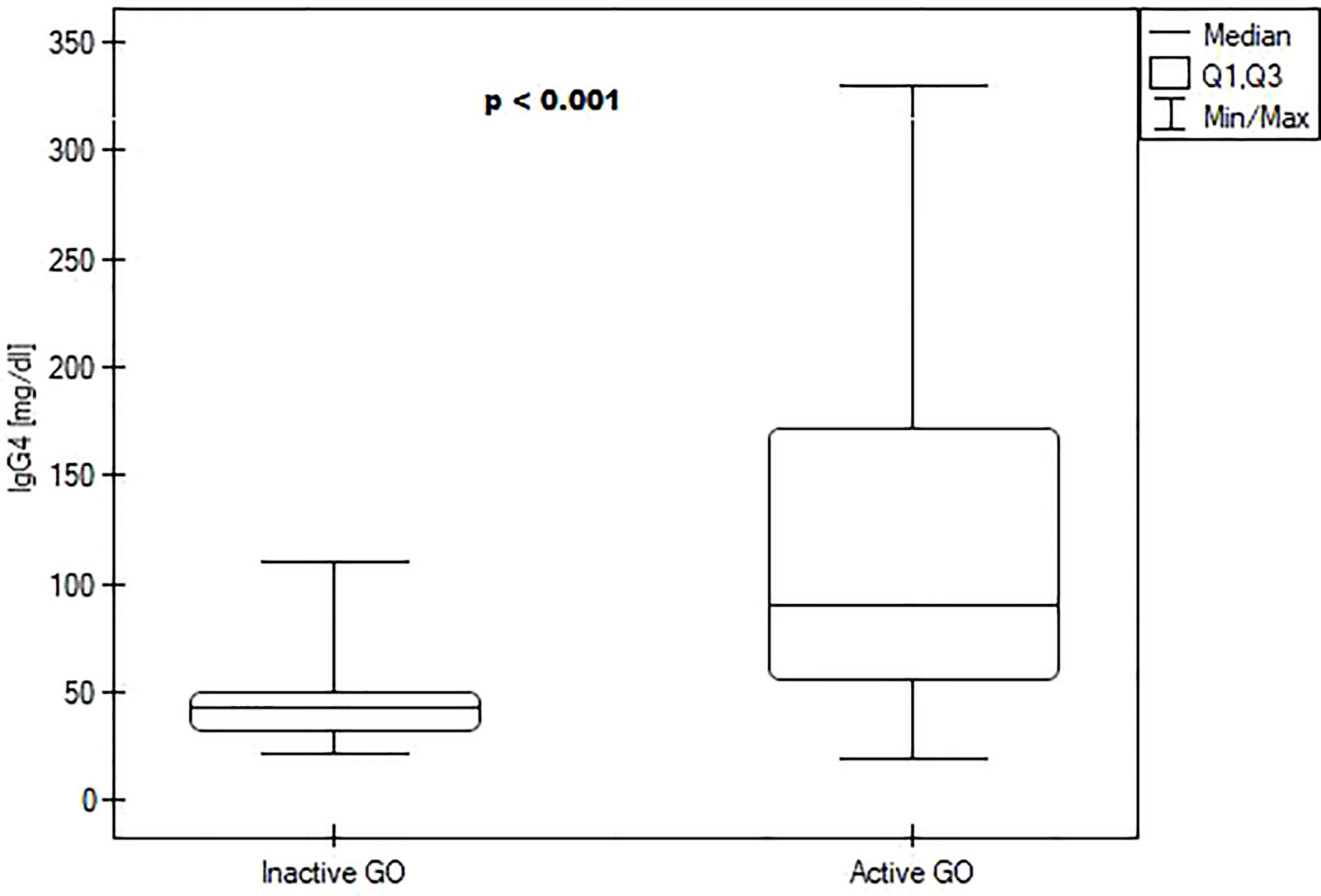
Boxplots comparing median IgG4 levels between the inactive Graves orbitopathy group and active Graves orbitopathy group.

We divided patients into groups based on the activity of the disease. Patients with active disease had a higher IgG4 level (89.95 (55.48; 171.1) vs 43.45 (32.48; 49.68) mg/dl, P<0.001) – **Figure 1**. They had also higher TRAb titers (13.79 (15; 20.7) vs 0,8 (0.54; 2.4) μU/ml, P=0.012. No differences in TSH, fT3, fT4, TPOAb, and TgAb were observed (**Table 4**). Patients in the active group also tended to have lower bilirubin levels than patients in the inactive group, but this result was not statistically significant (P=0.056). We did not find any correlations between IgG4 and thyroid volume, CAS, TSH, fT3, fT4, TPOAb, TGAb, TRAblevels, and other laboratory parameters.

**Table 4:** Comparison of chosen biochemical parameters between inactive GO and active GO groups.

Parameter	Inactive GO (n=16)	Active GO (n=44)	P value	
IgG4 (mg/dl)	43.45 [32.48; 49.68]	89.95 [55.48; 171.1]	**<0.001**
TSH (μU/ml)	0.8 [0.54; 2.4]	1.35 [0.45; 3.6]	0.686
fT3 (pmol/l)	4.3 [3.95; 4.83]	4.17 [3.51; 4.85]	0.532
fT4 (pmol/l)	17.92 [16; 20.25]	18.7 [15; 20.7]	0.747
TRAb (IU/I)	2.07 [0.96; 3,9]	9.66 [2.47; 20.87]	**0.012**
TPOAb (IU/ml)	42 [12; 172]	66 [16; 238.5]	0.625
TgAb (IU/ml)	15 [13.5; 22]	31 [13; 643.5]	0.260
Bilirubin (mg/dl)	0.74 ± 0.25	0.60 ± 0.22	0.056

TSH, thyroid stimulating hormone; fT3, free triiodothyronine; fT4, free thyroxine; TRAb, anti-TSH receptor antibodies; TPOAb, anti-thyroid peroxidase antibodies; TgAb, anti-thyroglobulin antibodies; IgG4m Immunoglobulin 4. Continuous variables with a normal distribution are shown as mean ± SE, nonparametric variables are shown as median [lower quartile, upper quartile], nominal variables are snown as N(%). Statistically significant P values (<0.05) were displayed in bold.

**Figure 2:**
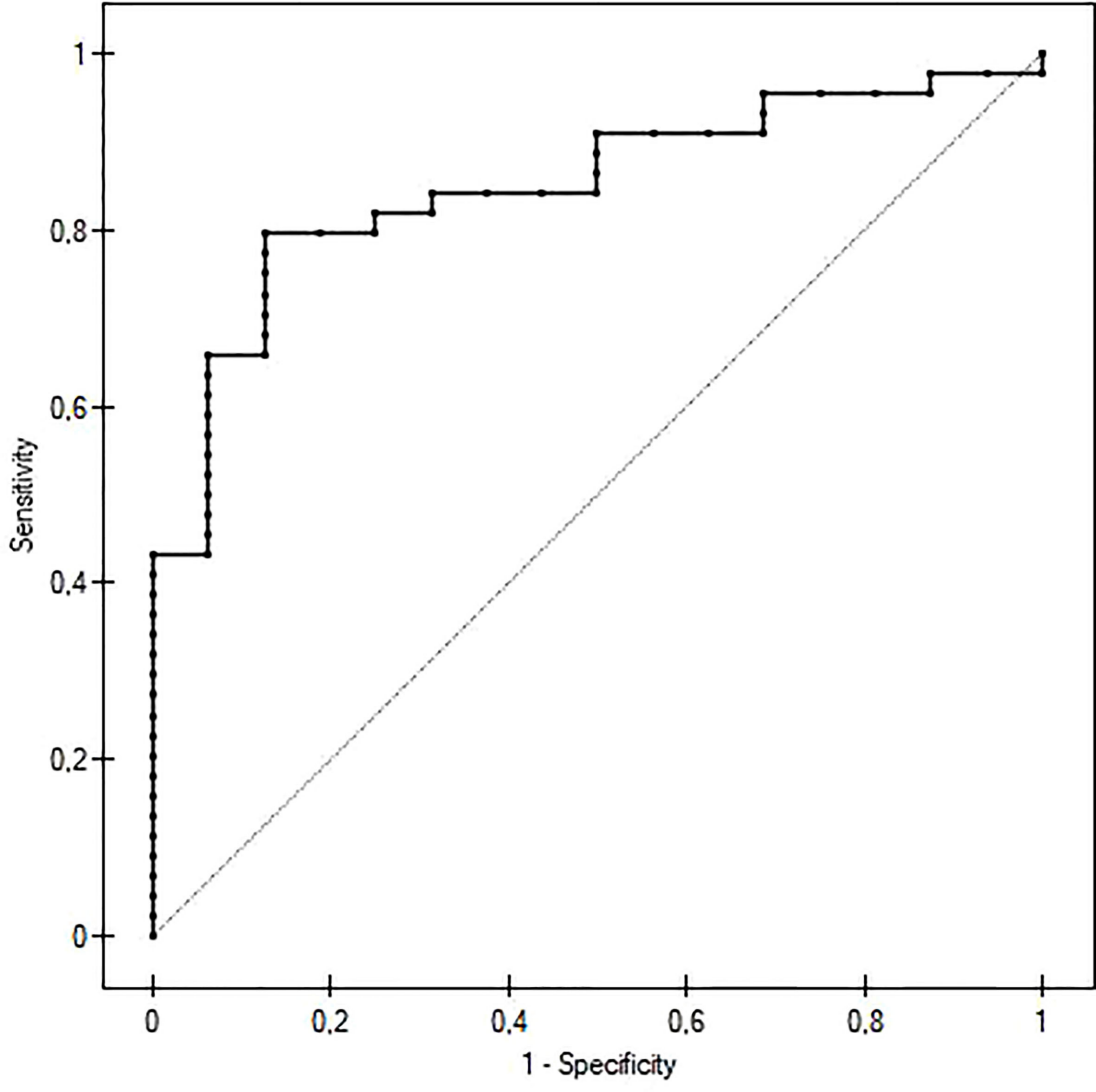
ROC curve of IgG4 serum concentration predicting active Graves orbitopathy. The cut-off value was 54,2 mg/dl. The AUC was 0,848 (P<0.001), sensitivity 79,5%, specificity 87,5%, positive predictive value 94,6%, negative predictive value 59,1%.

**Figure 3:**
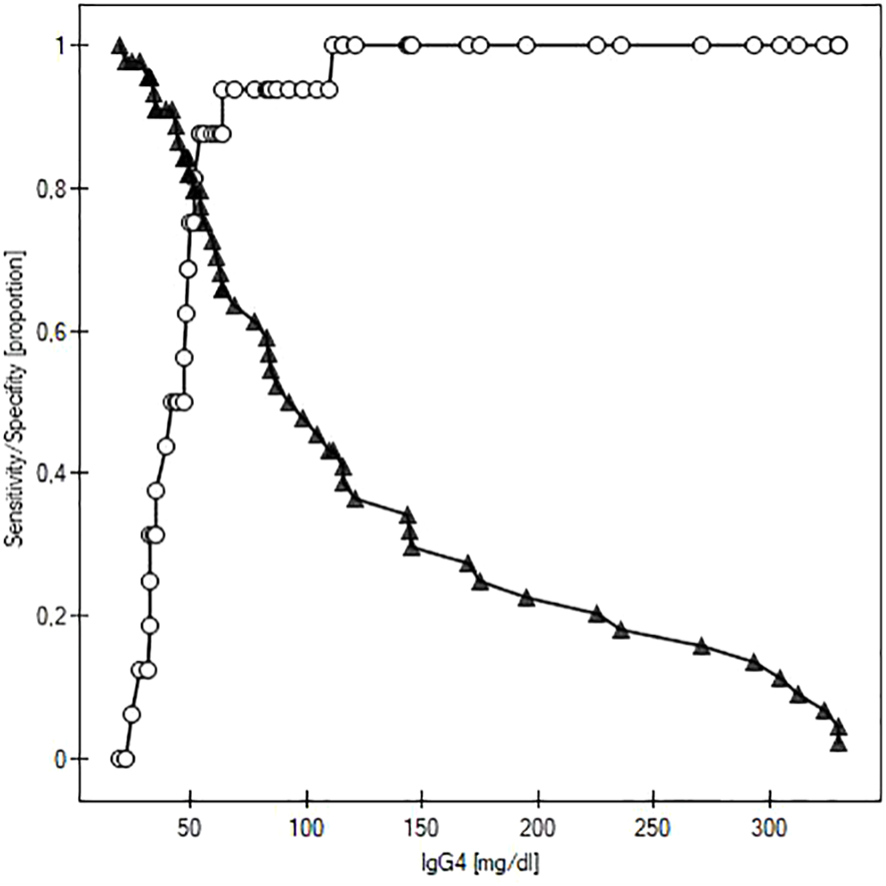
Curves of sensitivity (white circles) and specificity (black triangles) of IgG4 as a predictor of active Graves orbitopathy.

We performed a receiver operating characteristic (ROC) analysis to evaluate the potential of IgG as a diagnostic marker of GO activity (**Figure 2**). The cut-off for the maximum potential effectiveness of IgG4 as a biomarker (The Youden index) was ≥ 54.2 mg/dl. The AUC was 0.848 (P<0.001). For the aforementioned cut-off value sensitivity was 79.5%, specificity 87.5%, positive predictive value (PPV) 94.6%, negative predictive value (NPV) 59.1%. Sensitivity and specificity at different IgG4 serum concentrations are plotted in **Figure 3**.

## Discussion

Sixty patients with GO were enrolled in our study. 15 (25%) of our patients had IgG4 serum concentration elevated over 135 mg/dl, which is more prevalent than the reported average of IgG4 elevation in GO (17.6%) (14). Some authors, however, found high IgG4 levels in even 37.5% of GD patients (17). The mean IgG4 serum levels in both the high IgG4 group and normal IgG4 group were also similar to values reported by most previous authors (14).

In our study, 15 (100%) of the patients with high IgG4 levels had active GO compared to only 29 of 45 (64.44%) in the normal IgG4 group. Other studies have also reported a higher prevalence of active GO in elevated IgG4 patients (18, 19). Some studies have also found higher CAS in patients with elevated IgG4 titers (17, 19), while others failed to demonstrate any significant association between CAS and IgG4 levels (20–22).

However, our study is the only one where GO activity was assessed with the combined use of MRI with CAS. Other studies used only CAS to divide patients into active and inactive GO groups. Even though CAS is undoubtedly a great diagnostic tool, it has to be interpreted cautiously. Some papers report that its sensitivity can be as low as 55% and the PPV is 65% (23). Other researchers have compared the diagnostic utility of both CAS and MRI and found that MRI offers better sensitivity, specificity, PPV and NPV (24, 25).

Interestingly, in our study 8 out of 15 patients in the high IgG4 group had a CAS score of <3, but all of them had signs of active disease on MRI of the orbits. To a certain extent this could be explained by a small number of cases with severe or sight threating threating GO.

However, this also shows that we cannot rely solely on CAS in diagnosing active GO. There is a significant group of patients who present with troublesome symptoms (e.g., diplopia), which could still be treated with immunosuppressive/immunomodulatory drugs. If we relied solely on the CAS score, they might be falsely pronounced “inactive” and thus do not receive proper treatment. Based on this observation, we hypothesize that IgG4 might be a very sensitive marker of the inflammatory process, which is also elevated in patients, who do not show typical signs of active orbitopathy like inflammation of the conjunctiva, plica, eyelids, or chemosis.,but have active disease in the extraocular muscles and periorbital soft tissue. IgG4 serum concentration measurement would help to avoid false negative (“inactive”) diagnoses in patients with active disease and low CAS scores, especially in situations when access to MRI is limited, the patient has contraindications to MRI, or the results of imaging studies are ambiguous.

Various serum markers in GO have been studied recently, including Th2-derived chemokines (e.g., CCL2 or IL-29) (26, 27). Our ROC analysis confirms that IgG4 is a potential serum biomarker of active GO. It has a decent sensitivity and specificity with a very high PPV of 94.6% at the cut-off value of 54.2 mg/dl. This makes it a potentially very useful diagnostic test that could be used as a “rule in” test for active GO. test for active GO.

Some researchers described that patients with high IgG4 GD had high TRAb levels, which positively correlated with IgG4 serum concentration (18, 22). Others found increased TPOAb (19, 28) or TGAb levels (28). However, the majority of studies, including ours, did not find any statistically significant differences in TPOAb, TGAb (18, 20, 29), or TRAb (19, 20, 28, 29) between high IgG4 GD and normal IgG4 GD. Based on our study and the current body of evidence we would lean, towards the opinion that IgG4 is rather an independent marker of ongoing inflammation, without a particularly strong association with thyroid antibodies.

We did not find any associations between CAS and IgG4 levels, which is in concordance with results reported in other papers (20, 22). However, other research groups describe higher CAS levels in patients with high IgG4 GO (17, 19), and observed that IgG4 levels were ascending in order of CAS (18). The relationship between IgG4 levels and CAS remains equivocal; as demonstrated by Li et al, it seems to be significant in patients with a shorter duration of GO (19), and insignificant in patients with a longstanding disease, which comprised the majority of our group.

Patients with GD and elevated IgG4 levels were found to have lower echogenicity of the thyroid on ultrasound examination (20, 29). Unfortunately, the vast majority of our patient at the time of admission was already after thyroidectomy or radioiodine treatment. Thus, we were only able to compare thyroid volumes and could not perform a more detailed analysis of the ultrasound images.

A recent study by Li et al. reported that IgG4 levels decreased after intravenous methylprednisolone treatment and that patients with high IgG4 levels had better treatment outcomes. Our study was not longitudinal in design, and thus we could not check for post-treatment results. However, patients enrolled in our study, who were previously treated with methylprednisolone pulse therapy (>6 months ago) did not differ in IgG4 levels from treatment naïve patients.

Previous studies did not find any differences in ocular signs or symptoms other than CAS between patients with elevated IgG4 and normal IgG4 levels (19, 22). However, they did not report such a detailed ophthalmological examination as we did. Still, the only few significant differences we were able to find were a higher prevalence of lower eyelid retraction and a lower prevalence of glaucoma. There are no pathognomonic clinical features, which would differentiate GO from IgG4-RD, however eyelid retraction is associated rather with GO than IgG4-RD (30). We found a significantly higher eosinophil count in peripheral blood in high IgG4 GO patients compared to normal IgG4 GO. This finding is in concordance with the observations of Torimoto et al. (29) Interestingly, peripheral blood eosinophilia has also been reported in about 40% of patients with IgG4-RD (31, 32), however this similarity is insufficient to imply a direct correlation between those two diseases. Increased eosinophil count is also observed in patients with atopic conditions (33, 34)., but the prevalence of patients with atopic conditions in our cohort was small (4 in the normal IgG4 group, 0 in the high IgG4 group). This might be due to the exclusion criteria which were used in the study (we excluded patients with symptoms of infection, which overlap with those of exacerbated asthma or rhinitis, or patients during immunomodulatory treatment).

An interesting novel finding from our study is that patients in the elevated IgG4 group had lower bilirubin levels than GO patients with normal IgG4 values. Previous studies did not check for bilirubin levels. Historically, only high bilirubin serum concentrations were considered disturbing findings. However, more recently, low serum bilirubin levels have been reported as a risk factor for adverse outcomes like stroke or coronary artery disease, diabetes, cerebral deep white matter lesions, and many others (35–37). Bilirubin has antioxidative and anti-inflammatory properties. Preclinical studies on animal models showed that bilirubin could increase anti-inflammatory cytokine levels, suppress inflammatory cell recruitment and reduce the secretion of pro-inflammatory cytokines (38–40). Some studies suggest that bilirubin can be a protective factor for autoimmune diseases e.g., ulcerative colitis (41). Our results suggest that low bilirubin serum concentration might be associated with high IgG4 GO. Our study did not show that low bilirubin levels are associated with activity or severity of GO. Our results regarding bilirubin have to be interpreted with big caution as we have performed analyses of multiple parameters and thus the risk of a type I error is substantial. Applying a Bonferonni correction would render this result statistically insignificant. Further studies on this topic on larger groups with greater statistical power are warranted.

Our study has several limitations. Firstly, it was a single-center study. Secondly, most of the patients referred to our hospital had a history of the longstanding disease, and most of them had active GO, while patients with inactive GO were underrepresented. In addition, the majority of our patients were already after thyroidectomy or radioiodine treatment, and we had no histopathological data either from thyroid specimens or from orbital biopsies. We also did not assess total IgG serum concentration, however previous studies have shown that total IgG does not offer an adequate estimation of IgG4 levels.

## Conclusions

This study shows that patients with active GO have higher IgG4 serum concentrations than patients with inactive GO. Moreover, patients with high IgG4 GO have more frequently active GO, higher eosinophil count in peripheral blood, and lower bilirubin levels. We demonstrate that IgG4 serum concentrations can be used as a useful diagnostic tool to detect patients with active GO. Apart from disease activity, frequent lower eyelid retraction, and a decreased prevalence of glaucoma, we did not find many differences in clinical features between high IgG4 GO, and normal IgG4 GO. Thus, the debate if patients with high IgG4 GO are GO patients with particularly active disease or actually represent a distinct clinical entity related to IgG4-RD will probably continue until more studies, especially with histopathological examinations of thyroid specimens or orbital biopsies, emerge.

## Data availability statement

The raw data supporting the conclusions of this article will be made available by the authors, without undue reservation.

## Ethics statement

The studies involving human participants were reviewed and approved by Bioethics Committee at the Poznan University of Medical Sciences. The patients/participants provided their written informed consent to participate in this study.

## Author contributions

MO, ES-P, and MR contributed to the conception and design of the study. MO, AO-K, and PA organized the database. MO performed the statistical analysis. MO wrote the first draft of the manuscript. AO-K and PA contributed to the first draft of the introduction section. All authors contributed to manuscript revision, read, and approved the submitted version.
